# Effect of REDD+ projects on local livelihood assets in Keo Seima and Oddar Meanchey, Cambodia

**DOI:** 10.1016/j.heliyon.2020.e03802

**Published:** 2020-04-23

**Authors:** Sereyrotha Ken, Tomoe Entani, Takuji W. Tsusaka, Nophea Sasaki

**Affiliations:** a Graduate School of Applied Informatics, University of Hyogo, Japan; b Wildlife Conservation Society, Phnom Penh, Cambodia; c Natural Resources Management, Asian Institute of Technology, Thailand

**Keywords:** Business, Economics, Livelihood improvement, REDD+, Natural capital, Physical capital, Human capital, Financial capital, Carbon credit, Agricultural policy, Agroforestry, Ecological restoration, Forestry, Human geography, Natural resource management, Sustainable development, Ecosystem services, Biodiversity

## Abstract

Climate-change mitigation projects are expected to improve local livelihoods in targeted areas. Several REDD+ projects aimed at reducing emissions from deforestation and forest degradation, conserving and enhancing forest carbon stocks, and sustainably managing forests have been implemented in Cambodia but few studies have examined the effects on local livelihoods before and during project implementation. Our study applies a sustainable livelihood framework to assess the livelihood assets of local communities in the Oddar Meanchey and Keo Seima REDD+ project sites in Cambodia before and during project implementation. Five capital assets, namely natural, physical, human, financial, and social capital, are assessed and scored on a 1-to-5 Likert scale. Data analysis collected through 252 interviews in Oddar Meanchey and Keo Seima reveals a slight increase in livelihood assets in both sites from project validation to implementation. Generally, the mean scores for local livelihood assets increased from 2.81 ± 0.07 (±is followed by the standard error) and 2.66 ± 0.06 to 3.07 ± 0.09 and 3.06 ± 0.08 in Oddar Meanchey and Keo Seima, respectively. Nevertheless, natural capital assets sharply declined from 3.50 and 3.32 to 2.09 and 2.25, respectively. Respondents mainly blamed illegal logging for the decline, suggesting that strict patrolling and enforcement must be implemented. Furthermore, the scarcity of carbon-credit buyers and the projects’ inability to generate carbon-based revenues has led to dissatisfaction among local communities, inducing avoidable illegal activities in pursuit of short-term benefits. A financial mechanism to ensure sufficient and sustained financial support regardless of carbon-market volatility is urgently needed.

## 1 Introduction

Deforestation and forest degradation is still the second major source of global carbon emissions 12 years after the adoption of the Bali Action Plan of the United Nations Framework Convention on Climate Change (UNFCCC) in 2007 that adopted policy incentives to reduce carbon emissions from deforestation and forest degradation through conservation, sustainable management, and enhancement of carbon stocks, commonly known as the REDD+ (Reduce Emissions from Deforestation and forest Degradation by conserving forest carbon stocks, sustainably manage forests, and enhance forest carbon stocks) scheme. Recent studies have estimated that global deforestation emitted 4.0 PgCO2 year^−1^ during 2001–2010 and remained at 2.9 PgCO2 year^−1^ during 2011–2015 (Federici et al., 2015). Zarin et al. (2016) found similar emissions at 2.3 Pg CO2 year^−1^ between 2001 and 2013. Apart from carbon emissions, loss of forests reduces ecosystem services, especially the provisioning, supporting, and regulating services (Kim et al., 2018; Barrios et al., 2018) that 1.6 billion people depend on for daily subsistence and livelihood (Erbaugh and Oldekop, 2018). Foreseeing the consequences of deforestation and forest degradation, global leaders signed on to the Paris Climate Agreement and the Sustainable Development Goals in 2015 with both global agreements coming into force in 2016. Among the various strategies for implementing and achieving both agreements, REDD+ is an important mitigation option because of its ability to tackle climate change while safeguarding and improving local benefits and biodiversity (Phelps et al., 2012). Currently, there are 359 REDD+ active projects in the tropics (Simonet et al. 2019), although only about 300 have been actually implemented (Simonet et al., 2014).

However, the long-term sustainability of these REDD+ projects in mitigating climate change and safeguarding socioeconomic conditions and biodiversity remain questionable mostly because of low demand for carbon offsets from the projects (Laing et al., 2016; Foster et al., 2017) and lack of specific biodiversity goals (Panfil and Harvey, 2015). Enrici and Hubacek (2018) found that the deforestation rate in Indonesia had neither decreased nor stabilized even though REDD+ had been implemented in 2007. Similar declines in forest cover were seen in Cambodia (MoE 2018) and Myanmar (Cho et al., 2017) even though these countries had actively participated in REDD+ projects. Milne et al. (2019) reviewed REDD+ projects in mainland Southeast Asia and argued that many of the projects created social conflicts and yet failed to address the drivers of deforestation and forest degradation. Some studies, however, have found improvement as a result of REDD+ projects. Simonet et al. (2019), for instance, analyzed data from interviews with 181 farmers in the Brazilian Amazon and found that the REDD+ project reduced deforestation by up to 50%. Using publicly available social and spatial data, Jagger and Rana (2017) found that early REDD+ interventions protected the rights of local communities in Indonesia. Atela et al. (2015) found that the REDD+ project in Kenya improved land rights and local people's willingness to protect the forest. Furthermore, based on reviews of 80 REDD+ projects, Panfil and Harvey (2015) found some improvement in biodiversity safeguards and related capacity building where REDD+ projects had been implemented within the past 10 years. Moreover, through analysis of links between an agricultural census and remote-sensing data on deforestation and forest degradation, Godar et al. (2014) found that REDD+ areas dominated by smallholders could be protected from fragmentation and degradation. Based on a number of governance indicators tracked in the Maderacre & Maderyja Madre de Dios Amazon REDD projects in southeast Peru, Pettenella and Brotto (2012) found that transparency and accountability needed to be carefully addressed if REDD+ projects are to be successfully implemented.

Although these studies have shed light on the development and implementation of REDD+ projects in the tropics, studies on the effects of project implementation on local livelihoods remain limited. In addition to reducing carbon emissions, successful implementation of the REDD+ projects provides benefits to forest-dependent communities through intensive low-carbon agricultural practices and employment generation in farming, ecotourism, and social enterprises (Peras et al., 2016; CBD and GIZ, 2011). Indigenous and local communities are considered to be key stakeholders in protecting forest ecosystems and supporting the long-term efforts of REDD+ projects (CBD and GIZ, 2011). Local communities, especially indigenous people and forest-dependent communities play a crucial role in protecting and managing forest resources and their ecosystems. Properly designed, REDD+ activities can provide huge non-carbon benefits to locals (Hvalkof, 2013). Nevertheless, not all REDD+ projects have produced the expected results. In the Babati district in north-central Tanzania, Jacob and Brockington (2017) found that local communities were not satisfied with the REDD+ project's benefit-sharing because they perceived that weak governance resulted in many benefits going to a small group of elites.

Cambodia has suffered from deforestation and forest degradation for years. Studies on REDD+ and its implementation focusing on various aspects of REDD+ implementation from local to national levels have gained attention in recent years. Sasaki et al. (2016) examined the establishment of the forest reference emission level (FREL) while Nhem et al. (2017) focused on the use of media to improve the effectiveness of the REDD+ policy. Nathan and Pasgaard (2017) analyzed the contributions of the REDD+ project in Oddar Meanchey province to the economic efficiency, environmental effectiveness, and social equity of local communities. They found that carbon revenues from the carbon market alone would not be adequate to realize the REDD+ objectives of improving local livelihoods.

Presently, four REDD+ projects in Cambodia have been validated and implementation of these projects is underway. However, no study on the effects of REDD+ projects on local livelihood assets exists to guide future informed decision-making. This study aims to assess the livelihoods of local people living in two REDD+ project sites in Oddar Meanchey and Ratanakiri provinces using the sustainable livelihood framework. Local livelihoods are assessed in terms of five dimensions of capital assets — natural, physical, financial, social, and human — based on a five-point Likert scale.

## 2 Materials and methods

All procedures in the field survey and interviews with respondents were performed according to research standards followed at the Graduate School of Applied Informatics, University of Hyogo, in Kobe, Japan.

### 2.1 Update on the REDD+ projects in Cambodia

The Royal Government of Cambodia submitted its FREL report to the UNFCCC in 2016, which was later approved. The FREL is updated to reflect changes as data becomes available. Based on Cambodia's Ministry of Environment (2018) report (MoE, 2018), forest cover in Cambodia declined sharply from 57.5% in 2010 to 46.9% in 2014 and further to 45.0% in 2016 (Table 1). Cambodia lost about 2.65 million hectares of forest between 2006 and 2016, representing an annual decrease of about 2.45%.

**Table 1:** Forest cover changes in Cambodia between 2006 to 2016.

Classification	2006		2010		2014		2016	
	Ha	%	Ha	%	Ha	%	Ha	%
Evergreen Forest	3,710,271	20.4%	3,573,925	19.68%	2,973,903	16.38%	2,861,233	15.76%
Semi-evergreen Forest	1,453,441	8.00%	1,391,117	7.66%	1,108,320	6.10%	1,071,947	5.90%
Deciduous Forest	4,613,417	25.40%	4,498,397	24.77%	3,480,532	19.17%	3,336,349	18.37%
Flooded Forest	597,355	3.29%	524,005	2.89%	481,078	2.65%	477,813	2.63%
Forest Regrowth	216,123	1.19%	249,341	1.37%	228,560	1.26%	196,842	1.08%
Bamboo	129,837	0.71%	130,930	0.72%	130,678	0.72%	125,398	0.69%
Mangrove	32,060	0.18%	31,443	0.17%	33,002	0.18%	31,226	0.17%
Rear mangrove	27,519	0.15%	27,371	0.15%	25,906	0.14%	25,906	0.14%
Pine Forest	8,157	0.04%	8,157	0.04%	8,196	0.05%	8,195	0.05%
Pine Plantation	0	0.00%	11	0.00%	3,709	0.02%	3,870	0.02%
Tree Plantation	43,547	0.24%	17,214	0.09%	44,289	0.24%	43,122	0.24%
**Forest Area**	**10,831,727**	**59.64%**	**10,451,911**	**57.55%**	**8,518,173**	**46.90%**	**8,181,901**	**45.05%**
Oil Palm Plantation	35	0.00%	5,055	0.03%	36,311	0.20%	51,276	0.28%
Rubber Plantation	78,148	0.43%	137,307	0.76%	484,316	2.67%	509,224	2.80%
Grassland	600,006	3.30%	473,281	2.61%	351,337	1.93%	341,132	1.88%
Aagriculture	1,000,634	5.51%	1,275,444	7.02%	2,787,413	15.35%	3,017,435	16.62%
Paddy Field	3,668,981	20.20%	3,859,452	21.25%	4,133,474	22.76%	4,221,407	23.24%
Rock	219	0.00%	668	0.00%	2,054	0.01%	1,100	0.01%
Sand	8,304	0.05%	10,459	0.06%	40,581	0.22%	41,245	0.23%
Built up area	37,435	0.21%	43,800	0.24%	328,820	1.81%	352,987	1.94%
Village	248,126	1.37%	296,513	1.63%	42,166	0.23%	42,930	0.24%
Water	438,410	2.41%	458,658	2.53%	813,839	4.48%	783,849	4.32%
Wood shrub	1,248,649	6.88%	1,148,126	6.32%	622,190	3.43%	616,177	3.39%
**Non-forest**	**7,328,947**	**40.36%**	**7,708,763**	**42.45%**	**9,642,501**	**53.10%**	**9,978,762**	**54.95%**
**Total Area**	**18,160,674**	**100.00%**	**18,160,674**	**100.00%**	**18,160,674**	**100.00%**	**18,160,674**	**100.00%**

Four REDD+ projects have been validated by the Verified Carbon Standard (VCS) in Cambodia: community forests in Oddar Meanchey province (OM-REDD+ hereinafter); community forests in Keo Seima Wildlife Sanctuary of the Mondulkiri province (KS-REDD+); Tumring community forests in Kampong Thom province; and the most recent, the Southern Cardamom REDD+ Project in the Southern Cardamom National Park and Tatai Wildlife Sanctuary. OM-REDD+ has received three gold distinctions for climate, community, and biodiversity benefits while KS-REDD+ received a gold distinction for biodiversity benefits. In addition to these four projects, 13 other REDD and REDD+ projects are at various stages as shown in Table 2.

**Table 2:** Current status of REDD and REDD+ initiatives in Cambodia.

No.	Names of REDD+ Initiative	Responsible Authority∗	Current Status	Annual Reductions reported on VCS Project Database
1.	Oddar Meanchey Community Forest REDD+ Pilot Project (Project ID 904 in VCS Project database)	Forestry Administration (FA)	Validated by VCS and CCBA in October 2012 Verified in June 2014	204,792 CO_2_
2.	Reduced Emissions from Deforestation and Degradation in Keo Seima Wildlife Sanctuary (Project ID 1650)	Ministry of Environment (MoE)	Validated by VCS and CCBA in May 2017 Verified in May 2017	1,426,648 CO_2_
3.	Southern Cardamom REDD+ Project (Project ID 1748)	MoE	Validated in March 2018 by VCS Verified in December 2018	3,867,568 CO_2_
4.	Tumring REDD+ Project (Project ID 1689)	FA	Validated by VCS in June 2018	378,434 CO_2_
5.	Central Cardamom Mountains	FA	Unknown	
6.	Cardamom Mountains REDD+ Project	FA	Unknown	
7.	Siem Reap REDD Project	FA	Unknown	
8.	Prey Lang REDD Project	FA	Under validation for Joint Crediting Mechanism between Japan and Cambodia	
9.	Western Siem Pang Important Bird Area	FA	Unknown	
10.	Samlout REDD+ Project	FA-MoE	Unknown	
11.	Kulen Promtep Wildlife Sanctuary REDD+ Pilot Project	MoE	Unknown	
12.	Phnom Oral REDD+ Project	MoE	Unknown	
13.	Phnom Samkos REDD Project	MoE	Unknown	
14.	Lomphat Wildlife Conservation Area	MoE	Unknown	
15.	Koh Kong Mangrove and Flooded Forest REDD Project	Fisheries Administration (FiA)	Unknown	
16.	Kampong Chhang REDD Project	FiA	Unknown	
17.	Sihanouk Ville REDD Project	FiA	Unknown	

∗ The responsible authority may have changed after national elections in 2018 as some lands were reallocated to different ministries.

### 2.2 Description of the study sites

**Figure 1:**
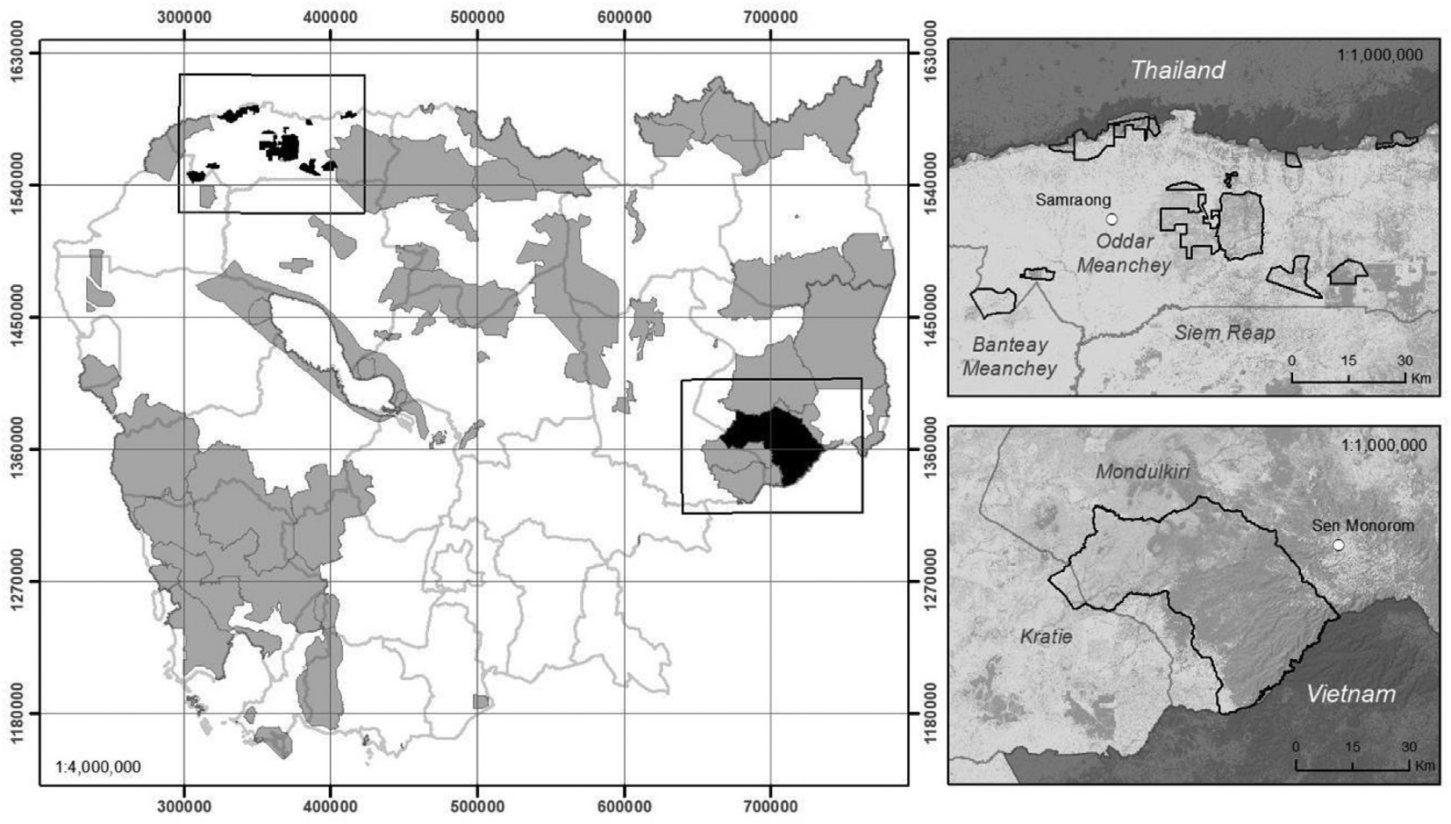
Location maps of OM-REDD+ (top) and KS-REDD+ (bottom) sites.

OM-REDD+ and KS-REDD+ are selected as study sites because project implementation had already been undertaken and subsequently verified in October 2012 and November 2015, respectively. OM-REDD+ is located in the province of Oddar Meanchey in northwestern Cambodia (Figure 1). OM-REDD+ was approved in 2007 by the Cambodian government and its validation period was extended from 2007 to 2012, when it was then validated by the Climate, Community, & Biodiversity Alliance (CCBA) and the VCS. OM-REDD+ is a 30-year project that began on Feb. 28, 2008, and is expected to last until Feb. 28, 2037. It consists of 13 community forests with a combined area of 63,831 ha, 56,050 of which are covered by forests (Terra Global Capital, 2012). OM-REDD+ is expected to generate an estimated 6,143,767 verified carbon units over 30 years.

KS-REDD+ is located in eastern Cambodia, mainly in Mondulkiri Province and partially in Kratie Province. The total area covers 166,983 ha in the Seima Protected Forest. KS-REDD+ consists of 20 villages in three districts (Keo Seima, Orang, and Sen Monorom) (Figure 1) (WCS, 2014). This project was approved in 2008, validated by the CCBA and VCS in November 2015, and verified by the VCS in April 2017. The project is expected to last until Dec. 31, 2069, and reduce carbon emissions of 14,266,485 tCO2e from 2010 to 2020.

To reduce the drivers of deforestation and forest degradation and thus reducing carbon emissions, various activities have been undertaken in both locations. Based on the Project Designed Documents for both REDD+ projects uploaded on the VCS website (https://www.vcsprojectdatabase.org), OM-REDD+ proposed to undertake nine activities while KS-REDD+ proposed to implement six activities (Table 3).

**Table 3:** REDD+ activities in both locations as listed in the Project Design Document.

OM-REDD+	KS-REDD+
1. Reinforcing of land-tenure status	1. Develop, approve, and implement legal and planning documents to reduce forest and wildlife crime through direct law enforcement
2. Sustainable forest and land-use planning	2. Establish sustainable community use of land and natural resources to adapt to climate change
3. Forest protection	3. Support alternative livelihoods that reduce pressure on forest and natural resource
4. Assisted natural regeneration and enrichment planting	4. Effective monitoring
5. Fuel-efficient stoves	5. Effective administration
6. Livestock protection from mosquitoes	6. Fund-raising
7. Agricultural intensification	
8. Natural resource management projects	
9. Fire prevention	

### 2.3 Data collection

This study collects and analyzes primary data. Field collection was undertaken in September and November 2018 in OM-REDD+ and KS-REDD+, respectively, using a mixed methods approach (Orr et al., 2016). Quantitative data were collected through a household (HH) survey while qualitative information was collected through key interviews. The questions used in the survey were modified from Qian et al. (2017) who studied on local livelihood under different governances of tourism development in Huangshan mountain area in China. However, some indicators and criteria used in their study were removed, altered or added to fit to the situation and the characteristic of the study sites. Heads (husband or wife who has influential decision and who generate more incomes for the family) of the HH were the intended respondents, however spouses were interviewed when the HH head was not available. Survey respondents and interview participants were asked to recall their livelihoods prior to project implementation (the period prior to verification) and during project implementation. The recall method has limitations because of potential inaccuracies of past memories. Nevertheless, it still provides useful information and tends to be reliable when the questions are closely related to livelihoods and daily activities (Nakano et al. 2018).

The minimum sample size for the HH survey was obtained using Yamane (1973) formula:

(1) $$n=\frac{N}{1+Ne^{2}}$$

where *n* = suggested minimum sample size for the HH survey in each REDD+ site; *N* = total household population in each site; and *e* = accepted margin of error (set at 10% or 0.10; i.e., 90% confidence level).

Since the HH population in OM-REDD+ was 9,893 HHs, Eq. (1) indicated a minimum sample size of 99 HHs. To allow for missing or erroneous observations in data, an additional 21 HHs were covered for a total sample size of 120 HHs. Likewise, the minimum sample size for KS-REDD+ with an HH population of 2,825 was calculated to be 97 HHs and 15 HHs were added for a total sample size of 112 HHs. In terms of interviews, one to three leaders per community forest participated in addition to six non-governmental organization (NGO) staff members and government officials. The breakdowns of sample size for the survey and interviews are presented in Table 4. These community forests were selected because they had implemented the REDD+ project activities since the projects were validated.

**Table 4:** Sample size for the HH survey and key interviews, 2018

Project	Community Forest	Key Interviews		HH Survey
		Key Participants	*n*	*n*
*OM-REDD+*	Sorng Roka Vorn	Leader	1	14
	Samaky	Leader	1	13
	Prey Srors	Leaders	3	38
	Rolus Thom	Leader	1	12
	Dung Beng	Leader	1	43
	Ratanak Ruka	Leader	1	0
		NGO staff	1	
	Total		8	120
*KS-REDD+*	Chakchar	Leader	1	35
	Anduong Kraloeng	Leaders	2	37
	Pu Char	Leaders	2	20
	Sre Preah	Leader	1	20
		NGO staff & government officials	5	
	Total		11	112

We employed the random sampling method for the HH survey to generate a representative sample of the population. However, not all residents were at home when the surveys were conducted. In those cases, an aspect of convenience sampling (Etikan et al., 2016) was adopted. Key interview participants were selected by the purposive sampling technique (Valerio et al., 2016) that is effective in targeting the most relevant respondents and thus the most pertinent information.

### 2.4 Assessment of the local livelihood assets or capital assets

Our study adopts a sustainable livelihoods framework (SLF) (Scoones, 1998) to assess the capital assets in the two sites. Under an SLF, local livelihood assets are defined as tangible and intangible goods and services owned and used by households or communities for daily subsistence and living. These capital assets represent five broad categories, namely natural, financial, human, social, and physical assets. Atela et al. (2015) employed an SLF to investigate the impacts of REDD+ projects on local livelihood assets in Taita-Taveta county in Kenya. Qian et al. (2017) used the SLF to understand the local livelihood assets under two ecotourism development systems in rural areas of China. An SLF is used in this study because of its ability to capture the complexities of local livelihoods, especially in rural areas (Scoones, 2009).

The five capital assets were assessed based on various indicators, criteria, and principles as shown in Table 5. Both households and key interviewees were asked to rate their perceptions of various indicators during the project validation and project implementation periods on a 1-to-5 Likert scale ranging from 1 (low) to 5 (high). The validation periods for OM-REDD+ and KS-REDD+ were before 2008 and before 2010, respectively. Local perceptions during the REDD+ project implementation period were the local perceptions that had formed prior to our fieldwork in 2018.

**Table 5:** Principles, criteria, and indicators for assessing the five capital assets.

Capital Assets	Principle Description	Criterion for Individual Principles	Indicators
Natural Capital	• Options for future use are maintained • Quality and quantity of natural resources and services are maintained or improved	• Biodiversity is conserved or not • Ecosystem function is maintained or not	• Biodiversity • Forest coverage • Environmental conservation
Physical Capital	• Physical capital is maintained or improved over time	• House physical status is maintained or improved	• Household fixed assets
Human Capital	• Ability to provide added value is improved over time	• Education or skill knowledge is improved or not • Local people's physical condition is maintained or improved	• Technical assistance • Environmental education • Skill and knowledge • Capacity building
Financial Capital	• Financial capital grows and is equitably distributed • Financial capital is circulated within the system	• Revenue is improved or not • Household harvest	• Household income related to forest • Household income not related to forest • Agricultural production
Social Capital	• Maintenance of systems of social reciprocity	• Economic and other shocks are buffered by system of social activity	• Rights in resource management/control over resources • Participate in community affair

It was difficult, and occasionally impossible, to collect information on income directly generated from the forests during our fieldwork because of the sensitivity on forest incomes and illegal logging. Locals were wary of answering freely and we therefore removed indicators of income both related to and not related to forest from our analysis.

### 2.5 Analysis

Descriptive statistics such as mean and frequency distribution are used to present respondents’ profiles. Mean values are also calculated for indicators of livelihood asset holdings. In general, it is controversial to compute means for ordinal-scale data such as Likert-scale data (Michell, 2014). In our study, however, most of the important analyzed indicators are based on multi-item sub-measurements and thus can generate more than 30 possible outcomes. Therefore, this study treats the main indicators as a semi-continuous measurement for which mean values are presented. An overall indicator is defined per livelihood capital category (e.g., financial, natural, etc.) as the mean over the sub-indicators in each category. The aggregate indicator is the mean over the five overall indicators.

As for inferential analysis, the Wilcoxon signed rank test (Seetha et al., 2019), a nonparametric alternative to the paired t test, is employed to examine the change in livelihood indicators over time. Furthermore, harnessing the recall data, the panel regression method (Tsusaka and Otsuka, 2013) is adopted to identify the change in livelihood indicators while determining and controlling for factors affecting their levels. Moreover, in the panel regression the difference-in-difference framework (Seetha et al., 2018) is incorporated to estimate the differential effects between the two projects on the livelihood indicators.

## 3 Results

### 3.1 Socioeconomic characteristics of the respondents

The survey was conducted in 120 HHs in OM-REDD+ and 112 HHs in KS-REDD+. In both project areas, there were more female respondents than male respondents (Table 6) because the survey was conducted during rice-harvesting season when more men were in the paddy fields. The respondents’ ages ranged from 17 to 75 while most ranged from 22 to 60. All respondents were directly involved in some type of work to support the family. The majority of respondents (96% in OM-REDD+ and 92% in KS-REDD+) were married. Many of the HHs (68% in OM-REDD+ and 59% in KS-REDD+) had four to six members. In terms of education, 69% and 72% of respondents in OM-REDD+ and KS-REDD+ had completed primary school, respectively.

**Table 6:** Demographic profile of surveyed HHs.

Demographic variable	Category	OM-REDD+ (*n* = 120)		KS-REDD+ (*n* = 112)	
		Frequency	Percent	Frequency	Percent
Gender	Male	49	40.8	35	31.3
	Female	71	59.2	77	68.8
Age	17–30	22	18.3	42	37.5
	31–45	39	32.5	45	40.2
	46–60	42	35.0	18	16.1
	>60	17	14.2	7	6.3
Marital status	Single	2	1.7	6	5.4
	Married	115	95.8	103	92.0
	Divorced, Widow, or Widower	3	2.5	3	2.7
Number of HH members	1 to 3	26	21.7	14	12.5
	4 to 6	82	68.3	66	58.9
	More than 6	12	10.0	32	28.6
Completed education level	No education 1	37	30.8	29	25.9
	Literacy class 3	0	0.0	2	1.8
	Primary school 4	55	45.8	52	46.4
	Secondary school 5	20	16.7	17	15.2
	High school 6	7	5.8	12	10.7
	College or higher 8	1	0.8	0	0.0

### 3.2 Capital assets

#### 3.2.1 Natural capital

The mean overall score for natural capital stock in OM-REDD+ before project implementation was 3.50 ± 0.09 (mean ± standard error), which decreased to 2.09 ± 0.09 during implementation, a decline of 40% (Table 6). Similarly, the overall score for natural capital stock in KS-REDD+ declined by 32% from 3.32 ± 0.09 before implementation to 2.25 ± 0.09 during implementation. One possible cause is that early in the project when carbon-based revenues were initially priced, people were highly motivated to participate in the project. However, the validation period was lengthy for both project sites and by the time OM-REDD+ was approved in 2012 and KS-REDD+ in 2015, carbon markets had started to collapse (Fletcher et al., 2016), driven by a global failure to reach the highly anticipated climate agreement at COP15 in Copenhagen in 2009 (Bond, 2010). Since demand for carbon credits was low compared to excessive supply, many carbon credits generated from the REDD+ projects were untradeable (Peters-Stanley et al., 2013). This was particularly true in OM-REDD+, where despite having been verified with triple gold recognition, it could not sell the carbon credits it could generate. Personal communications with the government officer in charge of the project in Oddar Meanchey in 2018 and 2019 suggest that few of the verified carbon units (credits) have been sold to this point.

Based on the calculated changes of scores in natural capital, the KS-REDD+ project performed somewhat better than OM-REDD+ in biodiversity conservation and forest cover protection. This probably is due to its location in a more peaceful area where local communities, rangers, and NGOs can patrol and monitor the forests. The OM-REDD+ site, on the other hand, has been affected by border conflicts between Cambodia and Thailand from June 2008 to December 2011, when both countries mobilized and stationed soldiers along their borders. The collapse of carbon markets seems to have affected both locations equally. During the stakeholder consultative workshops, villagers were informed of the potential revenues from carbon credits if they gave up illegal logging and jointly protected the forests. However, since actual carbon revenues from the REDD+ projects were miniscule compared to what they had been told during workshops, villagers grew to distrust the project developers and resorted to illegal clearing or logging to meet the immediate needs of their families. Financial support also has been found to be the main challenge for the successful implementation of REDD projects in Tanzania (Scheba, 2018).

#### 3.2.2 Physical capital

The mean score for physical capital stock representing household fixed assets is 3.03 ± 0.12 in OM-REDD+ before REDD+, which then increased by 19% to 3.62 (Table 7). Similarly, the score for physical capital stock at KS-REDD+ rose from 2.56 ± 0.11 to 3.69 ± 0.12, an increase of 61%. Local communities agreed that during implementation, there was an increase in household fix assets, improvement of local utilities, and construction of infrastructure.

**Table 7:** Mean scores for natural capital assets in OM-REDD+ and KS-REDD+ project sites: sub-indicators and overall indicator.

Indicators	Before	During	WSR test (*p*-value)	Change (%)
OM-REDD+ project site (*n* = 120)				
Biodiversity	3.76	1.58	0.000	-58
Improvement in forest coverage	3.58	1.68	0.000	-53
Environmental conservation	3.17	3.05	0.782	-4
Overall	3.50	2.09	0.000	-40
KS-REDD+ project site (*n* = 112)				
Biodiversity	3.60	2.10	0.000	-42
Improvement in forest coverage	3.21	2.01	0.000	-37
Environmental conservation	3.14	2.65	0.000	-16
Overall	3.32	2.25	0.000	-32

Note: WSR test = Wilcoxon Signed-Rank test.

For OM-REDD+, the support for physical capital came from various sources and in different forms such as sanitary toilets and a water-cleaning system provided by Marileur and a water purification system by Sarmaritan. For KS-REDD+, carbon finance supported the construction of wells, meeting halls, and public infrastructure. Moreover, the WCS had provided REDD+ funding to build toilets for local communities. As well, the communities in KS-REDD+ received support from the Cambodian Rural Development Team (a local NGO) to clean the water system and toilet and from Sedak for ponds and toilets. With support from the different partners, the majority of households at the KS-REDD+ site now have toilets and clean water.

Although the project-implementation period thus far has been relatively short, the findings of an increase in physical capital stock (Table 8) are consistent with those of Atela et al. (2015) in Kenya, who found that REDD+ had improved community-level physical capital such as clinics and schools.

**Table 8:** Mean scores for physical capital assets (household fixed assets) in OM-REDD+ and KS-REDD+ sites.

Project site	*n*	Before	During	WSR test (*p*-value)	Change (%)
OM-REDD+	120	3.03	3.62	0.002	+19
KS-REDD+	112	2.56	3.69	0.000	+44

Note: WSR test = Wilcoxon Signed-Rank test.

#### 3.2.3 Human capital

Mean overall scores for human capital holdings in the OM-REDD+ site were 2.50 ± 0.05 before implementation and rose to 3.80 ± 0.05 during implementation, an increase of 52%. Likewise, the overall scores for human capital holdings in KS-REDD+ increased by 56% from 2.36 ± 0.06 before implementation to 3.67 ± 0.05 during implementation (Table 9).

**Table 9:** Mean scores for human capital asset holding in OM-REDD+ and KS-REDD+ project sites: sub-indicators and overall indicator.

Indicators	Before	During	WSR test (*p*-value)	Change (%)
OM-REDD+ project site (*n* = 120)				
Technical assistance	2.62	3.93	0.000	+50
Environmental education	2.33	3.94	0.000	+69
Skills and knowledge	2.38	4.02	0.000	+69
Capacity building	2.56	3.23	0.000	+26
Overall	2.50	3.80	0.000	+52
KS-REDD+ project site (*n* = 112)				
Technical assistance	2.46	3.86	0.000	+57
Environmental education	2.14	3.90	0.000	+82
Skills and knowledge	2.45	3.73	0.000	+52
Capacity building	2.32	3.08	0.000	+33
Overall	2.36	3.67	0.000	+56

Note: WSR test = Wilcoxon Signed-Rank test.

The results show that all indicators of human capital increased during project implementation. In both locations, the progress in environmental education was particularly pronounced, while the progress in capacity building was relatively slow. The environmental education indicator achieved a higher score since during project formulation and development, local households receive training on different aspects of natural resources and environmental management through repeated consultative workshops. The workshops are forums to provide updated information, listen to farmers’ concerns, and propose REDD+ activities for implementation. Technical assistance and skills and knowledge indicators also achieved higher scores in both locations. Our results are consistent with those of previous studies that found positive effects of REDD+ projects on human capital in Kenya (Atela et al., 2015) and in the tropics based on a review of 45 articles (Duchelle et al., 2018). Where there were conflicts and natural disasters, human capital growth has tended to stagnate as the use of resources are focused on maintaining peace and stability.

As the demand for OM-REDD+ carbon credits became less attractive due to border conflicts and the collapse of carbon markets, carbon-based financial incentives were not available for human capital. Instead, the communities were supported by the SIDO for swine- and poultry-raising training and high-production rice and vegetable farming. The Cambodian Department of Women's Affairs also provided training on processing non-timber forest products (NFTP) for long-term storage. In addition, Prey Srors had a savings and rice bank where local farmers could borrow and deposit money. The rice bank allowed farmers to borrow rice and repay the borrowed amount in rice. This is found to be practiced and successfully implemented in Scheidel and Farrell (2015), Author links open overlay panel (Farrell and Silva-Macher, 2017). The rice bank can also help avoid potential conflicts of interests, especially when the price of rice varies season to season.

At KS-REDD+, carbon financing from the project helped provide capacity building for local committees. Thus, local communities were able to create their own three-year development plan. The committees held open meetings and identified areas that needed support such as clean water systems, wells, meeting halls, water holes, and bridges. However, the WCS has also provided agricultural training, especially for fruit growing in six villages, namely Pu Chram, Srae Prah, Ou Rona, Srae Lvea, Pu Char, and Ou Chrar.

#### 3.2.4 Financial capital

At the OM-REDD+ site, the mean overall indicator for financial capital asset holding increased by 24% from 2.04 ± 0.06 before REDD+ to 2.53 ± 0.06 during REDD+ (Table 10). Likewise, at the KS-REDD+ site, it increased by 31% from 1.09 ± 0.06 before REDD+ to 2.48 ± 0.06 during REDD+. The scores for financial capital are generally lower compared to the other types of livelihood assets. Particularly low is the sub-indicator for agricultural production with a mean of 1.37 and 1.63 in OM-REDD+ and KS-REDD+, respectively. Furthermore, the same sub-indicator did not improve from the pre-project period to the implementation period as shown by the result of the WSR test. In both sites, the indicator for forestry income exhibits higher values than the one for non-forestry income, which is understandable as both sites have substantial areas covered by forests. On the other hand, the sub-indicator for non-forestry income registered higher growth rates in both sites than the sub-indicator for forestry-related income.

**Table 10:** Mean scores for financial capital asset holding in OM-REDD+ and KS-REDD+ project sites: sub-indicators and overall indicator.

Indicators	Before	During	WSR test (*p*-value)	Change (%)
OM-REDD+ project site (*n* = 120)				
Household income related to forest	2.73	3.46	0.000	+27
Household income not related to forest	2.03	2.78	0.000	+37
Agricultural production	1.37	1.32	0.599	-4
Overall	2.04	2.53	0.000	+24
KS-REDD+ project site (*n* = 112)				
Household income related to forest	2.38	3.25	0.000	+37
Household income not related to forest	1.69	2.63	0.000	+56
Agricultural production	1.63	1.55	0.141	-5
Overall	1.90	2.48	0.000	+31

Note: WSR test = Wilcoxon Signed-Rank test.

Table 11 identifies the occupations workers in local communities were engaged in, whether as a main income source or as a supplement. Crop farming was the main occupation for 95% of HHs at OM-REDD+ and 85% of HHs at KS-REDD+. Second was livestock farming (50% of HHs at OM and 47% of HHs at KS), followed by NTFP harvesting (17% at OM and 27% at KS).

**Table 11:** Occupation of respondents in the REDD+ project sites: multiple responses.

Occupation	OM-REDD+ (*n* = 129)		KS-REDD+ (*n* = 123)	
	No. of Responses	Percent of *n*	No. of Responses	Percent of *n*
Crop farming	123	95	105	85
Livestock farming	64	50	58	47
NTFP harvesting	22	17	33	27
Forest ranger	31	24	12	10
Hunting	3	2	0	0
Fishing	16	12	12	10
Government job	7	5	16	13
Casual labor	13	10	7	6
Business	14	11	22	18
NGO jobs	0	0	5	4
Other occupation	10	8	10	8

Hvalkof (2013) and Poudel et al. (2015) found that REDD+ could contribute to maintaining sustainable livelihoods, food security, dynamic subsistence, income generation, and employment opportunities. Our findings in both locations confirm that REDD+ projects have contributed to maintaining sustainable livelihoods and food security. To achieve long-term sustainable development in both locations, greater emphasis should be placed on improving soil fertility, conserving underground water, and storing water for agricultural cultivation since the majority of locals are farmers who depend almost entirely on rainfall and soil fertility. In addition, as healthy forests can provide various ecosystem services to locals, REDD+ activities must urgently include restoration of degraded forests through planting, fire prevention, and prevention of unauthorized exploitation of fuelwood. Only 22 families (7.2% of respondents) in OM-REDD+ and 33 families (11.8%) in KS-REDD+ collected NTFP for their daily livelihood, either as direct or indirect sources of income. Of particular interest, only one family in each area was involved in NTFP collection as the main source of income. Therefore, forest products are not the main direct income source for local communities in either province.

#### 3.2.5 Social capital

At OM-REDD+, the mean overall indicator of social capital asset holding increased by 7% from 2.43 before REDD+ to 2.60 during REDD+ (Table 12). Likewise, at KS-REDD+ site, it increased by 11% from 2.28 to 2.52. These increases were statistically significant in both sites. The differences between the two sites in terms of social capital levels as well as their changes were generally minor. However, the scores varied widely across sub-indicators. The sub-indicators on Q40, Q41, and participation in community affairs had low scores while those on Q36 and control over resources showed relatively high scores. It is noteworthy that the sub-indicator for control over resources has registered no improvement since the REDD+ project began.

**Table 12:** Mean scores for financial capital asset holding in OM-REDD+ and KS-REDD+ project sites: sub-indicators and overall indicator.

Indicators	Before	During	WSR test (*p*-value)	Change (%)
OM-REDD+ project site (*n* = 120)				
Q34	2.09	2.57	0.000	23
Q36	3.50	3.12	0.004	-11
Q37	2.67	3.06	0.043	15
Rights in resources management/control over resources	3.69	3.70	0.617	0
Q39	1.82	1.83	0.638	1
Q40	1.65	1.70	0.046	3
Q41	1.62	1.67	0.177	3
Participate in community affairs	1.93	2.20	0.005	14
Q43	2.27	2.67	0.000	18
Q44	3.04	3.50	0.000	15
Overall	2.43	2.60	0.000	7
KS-REDD+ project site (*n* = 112))				
Q34	1.53	1.95	0.000	27
Q36	3.46	2.72	0.000	-21
Q37	2.53	3.50	0.000	38
Rights in resources management/control over resources	3.36	3.33	0.694	-1
Q39	1.92	2.38	0.000	24
Q40	1.72	1.95	0.004	13
Q41	1.78	2.09	0.000	17
Participate in community affairs	1.54	1.87	0.001	21
Q43	1.80	2.35	0.000	31
Q44	3.17	3.03	0.515	-4
Overall	2.28	2.52	0.000	11

Note: WSR test = Wilcoxon Signed-Rank test.

#### 3.2.6 Multivariate analysis of livelihood capital assets, REDD+ implementation, and respondents’ characteristics

Table 13 presents the result of the random effect regressions including all relevant factor variables. We consider the coefficient as statistically significant when the corresponding *p*-value is smaller than 0.10. The levels of physical, financial, social, and aggregate capital were higher in the OM-REDD+ site than in the KS-REDD+ site before project implementation. For instance, the aggregate capital score was higher in OM-REDD+ by 0.250 than in KS-REDD+ before implementation. In both sites, natural capital levels significantly decreased during implementation while all other types of capital including aggregate capital significantly increased. For instance, the physical capital score increased in OM-REDD+ by 0.583 and in KS-REDD+ by 1.125 during implementation. The aggregate livelihood capital score increased 0.205 faster in KS-REDD+ than in OM-REDD+.

**Table 13:** Random effect regression analysis of determinants of livelihood capital assets.

Independent Variable	Marginal effects of independent variables (*p*-value)					
	Natural Capital	Physical Capital	Human Capital	Financial Capital	Social Capital	Aggregate Livelihood Capital
Baseline difference: OM vs. KS	0.076 (0.702)	0.638 (0.012)	0.105 (0.375)	0.271 (0.039)	0.161 (0.066)	0.250 (0.001)
Difference in change: OM vs. KS	-0.334 (0.061)	-0.542 (0.016)	0.006 (0.957)	-0.092 (0.371)	-0.062 (0.310)	-0.205 (0.000)
Change in OM: During vs. Before 1)	-1.408 (0.061)	0.583 (0.016)	1.301 (0.000)	0.579 (0.000)	0.236 (0.000)	0.228 (0.000)
Change in KS: During vs. Before	-1.074 (0.000)	1.125 (0.000)	1.301 (0.000)	0.579 (0.000)	0.236 (0.000)	0.433 (0.000)
Livestock income 2): 1 if Yes, 0 otherwise	-0.282 (0.009)	0.225 (0.105)	0.067 (0.300)	0.025 (0.731)	0.159 (0.001)	0.039 (0.345)
NTFP income 2): 1 if Yes, 0 otherwise	-0.086 (0.501)	0.022 (0.894)	-0.005 (0.947)	0.233 (0.008)	-0.012 (0.840)	0.030 (0.536)
Ranger income 2): 1 if Yes, 0 otherwise	0.213 (0.109)	-0.147 (0.389)	-0.002 (0.980)	0.274 (0.002)	0.133 (0.030)	0.094 (0.062)
Hunting income 2): 1 if Yes, 0 otherwise	0.225 (0.105)	-0.949 (0.076)	0.123 (0.619)	-0.481 (0.090)	-0.109 (0.573)	-0.366 (0.021)
Fishery income 2): 1 if Yes, 0 otherwise	-0.111 (0.454)	0.257 (0.177)	0.160 (0.071)	-0.079 (0.432)	0.066 (0.341)	0.0584 (0.300)
Business income 2): 1 if Yes, 0 otherwise	-0.193 (0.161)	0.293 (0.098)	0.177 (0.031)	0.052 (0.581)	-0.014 (0.828)	0.0629 (0.229)
Seven other variables 3)	insig	insig	insig	insig	insig	insig
Wald χ^2^ (d.f. = 20)	225.12 (0.000)	87.82 (0.000)	631.81 (0.000)	152.50 (0.000)	96.62 (0.000)	186.52 (0.000)
R^2^	0.337	0.164	0.588	0.244	0.184	0.274

Notes: *n* (number of observations) = 464.

Number of respondents = 232.

1) The sum of “change in KS” and “difference in change (OM vs. KS).” The *p*-values presented are the lower of the two original coefficient *p*-values.

2) Dummy variables that take the value of one when the respondent has income from the respective source and zero otherwise.

3) Seven variables that were statistically insignificant (i.e., *p* > 0.10) for all six capital assets are not presented in the table though they are included in the analyses as control variables. “insig” stands for statistically insignificant. The seven variables are: respondent's sex, age, age squared, marital status, education level, and origin and whether the respondent worked as a civil servant, for an NGO, whether the HH had crop income, and family size.

Some respondent characteristics have significant effects on livelihood capital assets. Forest rangers had an aggregate capital level 0.094 higher on average than non-rangers while hunters had an aggregate capital level 0.366 lower on average than non-hunters. At a disaggregate level, livestock farmers tended to have lower levels of natural capital and higher levels of social capital than non-livestock holders. NTFP gatherers had higher levels of financial capital than non-gatherers. Fishers had higher levels of human capital than non-fishers. Those who ran their own business tended to have higher levels of physical and human capital. Finally, basic demographic variables such as age, sex, education, marital status, and family size have no significant effects on livelihood capital holdings.

## 4 Discussions

Although the overall scores have improved during the implementation period for both locations, some indicators have performed poorly (Tables 6, 7, 8, 9, 10, and 11). For example, the scores for access to forest management information, access to information on the implementation budget, and access to information on forest management planning have improved but remain below the neutral levels of 2.5. There are various reasons that could lead to these stagnant scores. Many community forests in Cambodia do not have forest management plans and a related budget. Even if written documentation exists, locals are often excluded from decision-making because they are illiterate or because they are not motivated to take management planning seriously unless there are monetary incentives. As information on REDD+ as a source of carbon-based income generation spread, local were motivated to learn more about issues such as budgets for forest management. However, since this information was not made available, local communities tended to develop negative perceptions. Previous studies (Husseini et al., 2016; Acheampong et al., 2018) have found that involving local communities in the planning of forest management activities can encourage active participation in project implementation and monitoring.

For both locations, the indicator “participate in any meeting for community or natural resources development and management” performs well (3.38 before, 4.11 during; with 3.81 and 4.25, respectively for both locations). This is probably due to the fact that since both locations are REDD+ project sites, more stakeholder consultations are required to have projects validated and verified.

**Figure 2:**
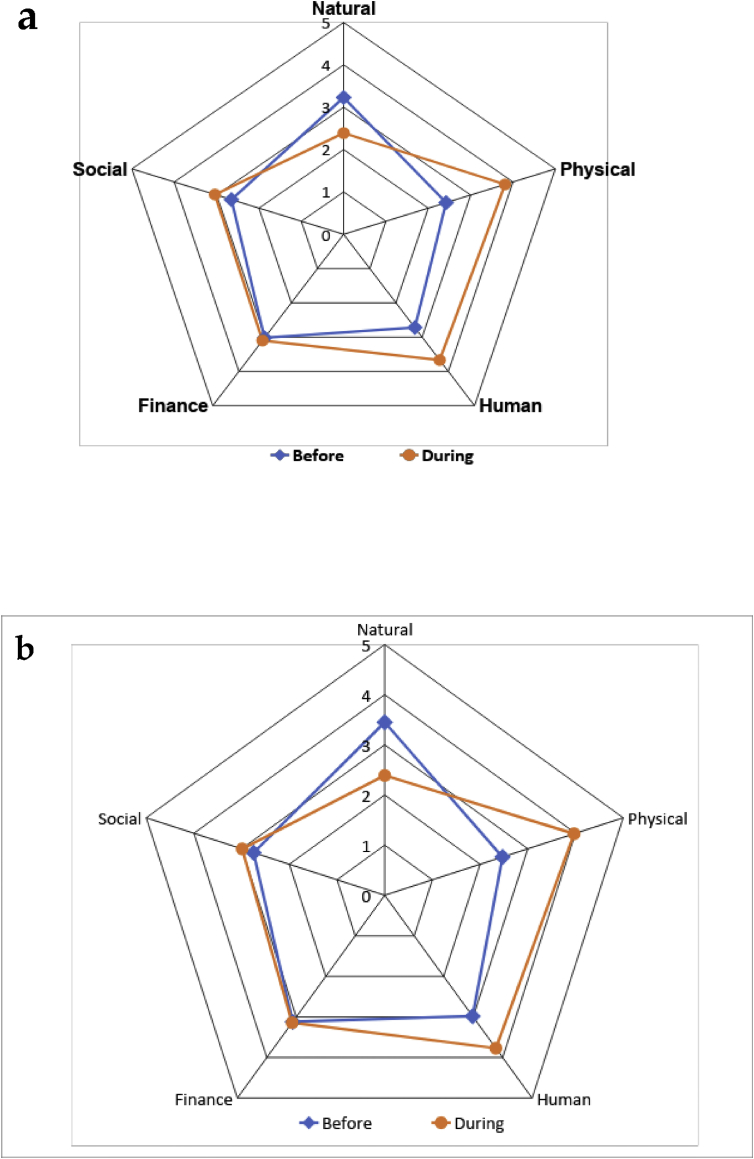
a. Local livelihood assets in KS-REDD+ before and during project implementation. b. OM-REDD+ and KS-REDD+ projects and local livelihood.

As REDD+ adds social value to forests, it can also maintain cultures and communities (Hvalkof, 2013). In both REDD+ areas, the scores for the social capitals of participation and decision-making are above average and improved during implementation. This is a positive sign that a REDD+ project benefits social capital (see Figure 2).

A main objective of OM-REDD+ activities regarding livelihood is agricultural intensification while for KS-REDD+ it is to support alternative livelihoods that reduce pressures on forests and natural resources. The proposed agricultural intensification is to be implemented in the Oddar Meanchey REDD+ community only if there is carbon financing. However, there is no carbon finance generated from REDD+ and the activity has not been implemented. In KSWS REDD+, with its full support from WCS as well as from carbon finance revenue (USD $2.6 million) in 2016, alternative livelihood activities such as training for vegetable growing and animal husbandry have been implemented.

At the OM-REDD+ site, local communities were initially motivated by carbon-based incentives for forest protection because it was the first in Cambodia. However, the inability of the REDD+ project developer (the forestry administration) to deliver carbon revenues as promised led to a loss of trust from local communities and has encouraged some to pursue a business-as-usual scenario.

At the KS-REDD+ site, local communities were not as interested in forest protection because they had heard about the inability of the project developer to deliver the promised revenues. Nonetheless, KS-REDD+ was able to generate carbon revenues and local communities received benefits in various forms. Therefore, local communities regained trust in the REDD+ project and are highly motivated.

Many local residents are illiterated and their trust and motivation depend mostly on the ability of the project developer to deliver on promises. Forestry has been a sensitive issue due to illegal logging and land clearing by both sides, local communities as well as government authorities. Carbon financing has played an important role in maintaining local involvement in project implementation at the KS-REDD+ site. Support from NGOs (i.e., WCS) during each step of implementation of REDD+ activities has contributed significantly to the success of KS-REDD+. Therefore, maintaining carbon financing for local communities is critical for the long-term success of REDD+ projects in Cambodia.

Our findings are in line with those of previous studies. Duchelle et al. (2018) reviewed 45 articles on REDD+ implementation and its impacts on local livelihoods and agreed that the lack of long-term financial support hampers the sustainability of REDD+ projects. To lessen dependence on carbon markets that are significantly affected by legislation and global agreements, financial support should be aimed at turning individual REDD+ activities (such as intensive farming, organic farming, fish farming, forestry enterprises, etc.) into investment projects either for locals or for which locals are hired and share in the benefits.

## 5 Conclusion

REDD+ projects are important performance-based financial incentives for reducing emissions from deforestation and forest degradation and for enhancing forest carbon stocks in developing countries. Using questionnaire data, this study assesses local livelihoods before and during implementation of REDD+ activities in two project sites in Cambodia. An SLF is adopted to assess the livelihood improvement according to 14 indicators of livelihood capital assets. In general, a significant increase in overall capital assets is seen in the two REDD+ sites. Specifically, physical capital asset achieved the highest rate of increase (about 57.4–60.7%) before and during implementation of REDD+ activities followed by human capital (26.5–34.9%). However, natural capital asset sharply declined by about 31% and 26% in the OM-REDD+ and KS-REDD+ sites, respectively. Lack of sustained carbon-based financial support created distrust among local communities and the project developer and consequently the status quo of illegal logging and land clearing for personal gain remained and contributed to the decline of natural capital assets. It is essential that sustainable, performance-based financial support to reduce carbon emissions or improve carbon storage in forests is available and that benefit sharing is clear and transparent to gain trust and maintain participation from local communities.

Given the unpredictability of carbon-based revenues and volatile carbon markets, it is important to create alternative sources of income through various REDD+ project activities such as investment in sustainable agriculture, production of efficient cooking stoves, renewable energy for rural electrification, eco-tourism, and social enterprises for NTFP for online or offline sales. With sustainable income from any of these investment opportunities, local communities are likely to focus on forest protection. Therefore, investment in REDD+ activities with local involvement could generate higher yet independent incomes for local communities for generations.

The authors conclude that REDD+ project implementation can contribute to the improvement of local livelihoods. As both projects are still ongoing, further study on the progress of REDD+ project implementation and transparent benefit sharing could provide additional insights into REDD+ projects and local livelihoods.

## Declarations

### Author contribution statement

N. Sasaki: Conceived and designed the experiments; Analyzed and interpreted the data; Contributed reagents, materials, analysis tools or data; Wrote the paper.

T.W. Tsusaka: Analyzed and interpreted the data; Contributed reagents, materials, analysis tools or data.

S. Ken: Conceived and designed the experiments; Performed the experiments; Analyzed and interpreted the data; Contributed reagents, materials, analysis tools or data; Wrote the paper.

T. Entani: Conceived and designed the experiments; Contributed reagents, materials, analysis tools or data.

### Funding statement

This research did not receive any specific grant from funding agencies in the public, commercial, or not-for-profit sectors.

### Competing interest statement

The authors declare no conflict of interest.

### Additional information

No additional information is available for this paper.

## Appendix A Supplementary data

Appendix_Nophea Appendix_Nophea

## References

[bib1] Acheampong E.O., Agyeman K.O., Amponsah O. (2018). The motivation for community participation in forest management: the case of Sefwi-Wiawso forest district, Ghana. Int. For. Rev..

[bib2] Atela J.O., Minang P.A., Quinn C.H., Duguma L.A. (2015). Implementing REDD+ at the local level: assessing the key enablers for credible mitigation and sustainable livelihood outcomes. J. Environ. Manage..

[bib3] Barrios E., Valencia V., Jonsson M., Brauman A., Hairiah K.I. (2018). Contribution of trees to the conservation of biodiversity and ecosystem services in agricultural landscapes. Int. J. Biodiv. Sci. Ecosyst. Serv. Manag..

[bib4] Bond P. (2010). Maintaining momentum after copenhagen's collapse: seal the deal or “seattle” the deal?. J. Capit. Nat. Social..

[bib5] CBD, GIZ (Secretariat for the Conservation on Biological Diversity, & Deutsche Gesellschaft für Internationale Zusammenarbeit (giz) GmbH.) (2011). Biodiversity and livelihoods: REDD-plus benefits. Montreal and Eschborn: Secretariat for the Convention on Biological Diversity and Deutsche Gesellschaft für Internationale Zusammenarbeit (giz) GmbH.

[bib6] Cho B., Naing A.K., Sapkota L., Thaung L. (2017). Stabilizing and Rebuilding Myanmar’s Working Forests: Multiple Stakeholders and Multiple Choices. Bangkok, the Nature Conservancy and RECOFTC-The Center for People and Forests.

[bib7] Duchelle A.E., Simonet G., Sunderlin W.D., Wunder S. (2018). What is REDD+ achieving on the ground?. Curr. Opin. Environ. Sustain..

[bib8] Enrici A.M., Hubacek K. (2018). Challenges for REDD+ in Indonesia: a case study of three project sites. Ecol. Soc..

[bib9] Erbaugh J.T., Oldekop J.A. (2018). Forest landscape restoration for livelihoods and well-being. Curr. Opin. Environ. Sustain..

[bib10] Etikan I., Musa S.A., Alkassim R.S. (2016). Comparison of convenience sampling and purposive sampling. Am. J. Theor. Appl. Stat..

[bib11] Farrell K.N., Silva-Macher J.C. (2017). Exploring futures for Amazonia's Sierra del divisor: an environmental valuation Triadics approach to analyzing ecological economic decision choices in the context of major shifts in boundary conditions. Ecol. Econ..

[bib12] Federici S., Tubiello F.N., Salvatore M., Jacobs H., Schmidhuber J. (2015). New estimates of co2 forest emissions and removals: 1990–2015. For. Ecol. Manag..

[bib13] Fletcher R., Dressler W., Büscher B., Anderson Z.R. (2016). Questioning REDD+ and the future of market-based conservation. Conserv. Biol..

[bib14] Foster B.C., Wang D., Auld G., Cuesta R.M.R. (2017). Assessing audit impact and thoroughness of VCS forest carbon offset projects. Environ. Sci. Pol..

[bib15] Godar J., Gardner T., Tizado A., Pacheco E.J.P. (2014). Actor-specific contributions to the deforestation slowdown in the Brazilian Amazon. Proc. Natl. Acad. Sci. U.S.A..

[bib16] Husseini R., Kendie B., Agbesinyale P. (2016). Community participation in the management of forest reserves in the Northern Region of Ghana. Int. J. Sustain. Dev. World Ecol..

[bib17] Hvalkof S. (2013). Imperative for REDD+ Sustainability: Non-carbon Benefits, Local and Indigenous Peoples. Warsaw: IBIS, CARE, IWGIA.

[bib18] Jacob T., Brockington D. (2017). Learning from the other: benefit sharing lessons for REDD+ implementation based on CBFM experience in Northern Tanzania. Land Use Pol..

[bib19] Jagger P., Rana P. (2017). Using publicly available social and spatial data to evaluate progress on REDD+ social safeguards in Indonesia. Environ. Sci. Pol..

[bib20] Kim Y.S., Latifah S., Afifi M., Mulligan M., Burk S., Fisher L., Siwicka E., Remoundou K., Christie M., Lopze S.M., Jenness J. (2018). Managing forests for global and local ecosystem services: a case study of carbon, water and livelihoods from eastern Indonesia. Ecosyst. Serv..

[bib21] Laing T., Taschini L., Palmer C. (2016). Understanding the demand for REDD + credits. Environ. Conserv..

[bib22] Michell J. (2014). An Introduction to the Logic of Psychological Measurement.

[bib23] Milne S., Mahanty S., To P., Dressler W., Kanowski P., Thavat M. (2019). Learning from ‘actually existing’ REDD+ A synthesis of ethnographic findings. Conserv. Soc..

[bib24] MoE (2018). Cambodia forest Cover 2016.

[bib3a] Nakano Y., Tsusaka T.W., Aida T., Pede V.O. (2018). Is farmer-to-farmer extension effective? The impact of training on technology adoption and rice farming productivity in Tanzania. World Develop..

[bib25] Nathan I., Pasgaard M. (2017). Is REDD+ effective, efficient, and equitable? Learning from a REDD+ project in Northern Cambodia. Geoforum.

[bib26] Ngoun P. (2014). Sustainable Forest Governance in the Asia-Pacific Region: Has REDD+ Adequately Addressed Drivers of Deforestation and Forest Degradation? Development Research Forum Policy Brief No. X (Phnom Penh: Cambodia Development Research Institute).

[bib27] Nhem S., Lee Y.J., Phin S. (2017). Sustainable management of forest in view of media attention to REDD + policy, opportunity and impact in Cambodia. For. Pol. Econ..

[bib2a] Orr A., Homann-KeeTui S., Tsusaka T.W., Msere H.W., Dube T., Senda T. (2016). "Are there women’s crops"? A new tool for gender and agriculture. Develop. Pract..

[bib28] Panfil S.N., Harvey C.A. (2015). REDD+ and biodiversity conservation: a review of the biodiversity goals, monitoring methods, and impacts of 80 REDD+ projects. Conserv. Lett..

[bib29] Peras J.R., Juan P., Inoue M., Mohammed A.J., Harada K., Sasaoka M. (2016). The Sustainable livelihood challenge of REDD+ implementation in the Philippines. Environ. Nat. Resour. Res..

[bib30] Peters-Stanley M., Gonzalez G., Yin D. (2013). Covering New Ground State of the Forest Carbon Markets 2013.

[bib31] Pettenella D., Brotto L. (2012). Governance features for successful REDD+ projects organization. For. Pol. Econ..

[bib32] Phelps J., Freiss D.A., Webb E.L. (2012). Win-win REDD+ approaches belie carbon-biodiversity trade-offs. Biol. Conserv..

[bib33] Poudel M., Thwaites R., Race D., Dahal G.R. (2015). Social equity and livelihood implications of REDD+ in rural communities-a case study from Nepal. Int. J. Commons.

[bib34] Qian C., Sasaki N., Jourdain D., Minsun Kim S., Shivakoti G.P. (2017). Local livelihood under different governances of tourism development in China – a case study of Huangshan mountain area. Tourism Manag..

[bib35] Sasaki N., Chheng K., Mizoue N., Abe I., Lowe A.J. (2016). Forest reference emission level and carbon sequestration in Cambodia. Global Ecol. Conserv..

[bib36] Scheba A. (2018). Market-based conservation for better livelihoods? The promises and fallacies of REDD+ in Tanzania. Land.

[bib37] Scheidel A., Farrell K.N. (2015). Small-scale cooperative banking and the production of capital: reflecting on the role of institutional agreements in supporting rural livelihood in Kampot, Cambodia. Ecol. Econ..

[bib38] Scoones I. (1998). Sustainable Rural Livelihoods: a Framework for Analysis. http://www.ids.ac.uk/files/dmfile/Wp72.pdf.

[bib39] Scoones I. (2009). Livelihoods perspectives and rural development. J. Peasant Stud..

[bib40] Seetha A., Tsusaka T.W., Munthali W., Musukwa M., Mwangwela A., Kalumikiza Z., Manani T., Kachulu L., Kumwenda N., Musoke M., Okori P. (2018). How immediate and significant is the outcome of training on diversified diets, hygiene, and food safety? An effort to mitigate child undernutrition in rural Malawi. Publ. Health Nutr..

[bib41] Seetha A., Tsusaka T.W., Njoroge S.M.C., Kumwenda N., Kachulu L., Maruwo J., Machinjiri N., Botha R., Msere H.W., Masumba J., Tavares A., Heinrich J.M., Siambi M., Okori P. (2019). Knowledge, attitude and practice of Malawian farmers on pre- and post-harvest crop management to mitigate aflatoxin contamination in groundnut, maize and sorghum—implication for behavioral change. Toxins.

[bib42] Simonet G., Karsentym A., de Perthuis C., Newton P., Schaap B. (2014). REDD+ Projects in 2014: an Overview Based on a New Database and Typology.

[bib1a] Simonet G., Subervie J., Ezzine-de-Blas D., Cromberg M., Duchelle A.E. (2019). Effectiveness of a REDD+ project in reducing deforestation in the Brazilian Amazon. Am. J. Agric. Econ.

[bib44] Terra Global Capital (2012). Reduced Emssions from Deforestation and Degradation in Community Forests- Oddar Meanchey, Cambodia.

[bib45] Tsusaka T.W., Otsuka K., Otsuka K., Larson D.F. (2013). The declining impacts of climate on crop yields during the Green Revolution in India: 1972 to 2002. An African Green Revolution: Finding Ways to Boost Productivity on Small Farms.

[bib46] Valerio M.A., Rodriguez N., Winkler P., Lopez J., Dennison M., Liang Y., Turner B.J. (2016). Comparing two sampling methods to engage hard-to-reach communities in research priority setting. BMC Med. Res. Methodol..

[bib47] WCS (2014). Reduced Emissions from Deforestation and Degradation in Seima Protection forest, Cambodia. Phnom Penh: Wildlife Conservation Society.

[bib48] Yamane T. (1973). Statistics - an Introductory Analysis.

[bib49] Zarin D.J., Harris N.L., Baccini A., Aksenov D., Hansen M.C., Azevedo-Ramos C. (2016). Can carbon emissions from tropical deforestation drop by 50% in 5 years?. Global Change Biol..

