# Supplementary material for: Effect of REDD+ projects on local livelihood assets in Keo Seima and Oddar Meanchey, Cambodia
Source: Heliyon. 2020 Apr 23;6(4):e03802. doi: 10.1016/j.heliyon.2020.e03802 (PMC7184172; doi:10.1016/j.heliyon.2020.e03802)
Supplement: Appendix_Nophea [file mmc1.docx]

**Appendix**

Effect of REDD+ Projects on Local Livelihood Assets in Keo Seima and Oddar Meanchey, Cambodia

**No. …………**

Questionnaire Survey

****How long have you been living here? ………………………………… (if s/he has just moved here later than 2008, move to the next respondent)**

Date : ………………… Time:…………………. Name of interviewer:…………………….

Name of respondent :…………..………………..Village:………………………………….

Commune:……………………………………….District:………………………………….

Name of Community Forest: …………………………………Others:……………………..

**Part 1. Socioeconomic**

1. Sex: ☐ 1. Male ☐2. Female
2. Age: ………………….. Year olds
3. Marital status: ☐ 1. Single ☐ 2. Married ☐ 3. Divorced

☐ 4. Other (Specify:……………………………….)

1. Number of household (people) : ………………
2. Education level: ☐ 1. No education ☐ 2. Informal education at local pagoda

☐ 3. Literacy class ☐ 4. Primary school  5. Secondary school ☐ 6. High school ☐ 7. Diploma, Vocational Education

☐ 8. College or higher

1. Origin: ☐ 1. Khmer ☐ 2. Vietnamese-Cambodian ☐ 3. Chinese-Cambodian

☐ 4. Cham/ Muslim ☐ 5. Indigenous minority (specify………………….)

1. What is your occupation (in case respondent has more than one occupation write number 1 in the box for main occupation, and following by number 2 , 3 etc)?

☐ 1. Farmer  2. Livestock raiser  3. NTFPs collector

4. Forest Ranger  5. Hunter ☐6. Fisher man

7. Government officer  8. Labor worker

9. Business man/woman  10. NGOs Staff

11. Others (specify:…………… ……)

Length of involvement: 1...............................

2...................................

3……………...............

1. Your family income from main source ( write number 1 in the box for the main source of income, and following by 2, 3 etc.)

☐ 1. Farming (specify………………………………………………….)

2. Livestock raising (specify…………………………………………)

3. NTFPs collecting (specify…………………………………………)  4. Forest Ranging  5. Hunting ☐6. Fishing  7. Government paid  8. Labor work paid

9. Business (Selling…….. ………….) ☐ 10. Remittances (from relatives)

☐ 11. NGOs work paid  12. Others (specify:…………… …………….)

1. Do you want to earn more from farming activities? ☐ 1. Yes  2. No
2. Would you like to sell your products online if there is the platform for you?

☐ 1. Yes ☐ 2. No

1. Would you like to grow the crops according to what is order? ☐ 1. Yes ☐ 2. No
2. Are you willing to plant trees around your house or farmland if you get paid annually for doing so? ☐ 1. Yes ☐ 2. No
3. Would you like to provide homestay to tourists who visit your farm and get paid?

☐ 1. Yes ☐ 2. No

1. What is your source of energy right now? ……………………………………………
2. How much you pay for energy? ……………….monthly or ……………yearly
3. Are you willing to install solar panels on your rooftop to get the cheaper energy than what you pay now? ☐ 1. Yes ☐ 2. No

If yes, in what condition?

1. You pay for the installation of solar. (how much you are willing to pay?............)
2. You get the loan to install it, then pay it back monthly or yearly when you can sell the farming product/service)
3. Other option specify………………………………………………………………..

**Part 2. Driver and activities to address deforestation and forest degradation**

1. Are you a community forest member? ☐ 1. Yes ☐2. No
2. Have you used to participate in any activities of forest management and conservation?

☐ 1. Yes ☐2. No

If yes, please describe: ………………………………………………………………..

………………………………………………………………………………………………

1. Do you know about REDD+ ? ☐ 1. Yes ☐ 2. No

If yes, just ask them to briefly what they know and understand about REDD+.......... ……………………………………………………………………………………………………………………………………………………………………………………………………………………………………………………………………………………………………………………………………………………………

The drivers of forest loss in your region (in their community and surroundings)

To what extend do you agree that the following items are the drivers of deforestation and forest degradation in your region?

| **Drivers** | **REDD+** | **Strongly disagree (1)** | **Disagree (2)** | **Neutral**  **(3)** | **Agree**  **(4)** | **Strongly agree**  **(5)** |
| --- | --- | --- | --- | --- | --- | --- |
| 1. Commercial logging | Before | 1 | 2 | 3 | 4 | 5 |
|  | During | 1 | 2 | 3 | 4 | 5 |
| 1. Illegal logging | Before | 1 | 2 | 3 | 4 | 5 |
|  | During | 1 | 2 | 3 | 4 | 5 |
| 1. Forest fire | Before | 1 | 2 | 3 | 4 | 5 |
|  | During | 1 | 2 | 3 | 4 | 5 |
| 1. Economic land concessions | Before | 1 | 2 | 3 | 4 | 5 |
|  | During | 1 | 2 | 3 | 4 | 5 |
| 1. Encroachment | Before | 1 | 2 | 3 | 4 | 5 |
|  | During | 1 | 2 | 3 | 4 | 5 |
| 1. Conversion to settlement | Before | 1 | 2 | 3 | 4 | 5 |
|  | During | 1 | 2 | 3 | 4 | 5 |
| 1. Clearing forest for agriculture | Before | 1 | 2 | 3 | 4 | 5 |
|  | During | 1 | 2 | 3 | 4 | 5 |
| 1. Forest clearing for land sales | Before | 1 | 2 | 3 | 4 | 5 |
|  | During | 1 | 2 | 3 | 4 | 5 |
| 1. Timber harvesting for domestic use | Before | 1 | 2 | 3 | 4 | 5 |
|  | During | 1 | 2 | 3 | 4 | 5 |
| 1. Fuelwood gathering | Before | 1 | 2 | 3 | 4 | 5 |
|  | During | 1 | 2 | 3 | 4 | 5 |
| 1. Improved road access | Before | 1 | 2 | 3 | 4 | 5 |
|  | During | 1 | 2 | 3 | 4 | 5 |
| 1. Population growth | Before | 1 | 2 | 3 | 4 | 5 |
|  | During | 1 | 2 | 3 | 4 | 5 |
| 1. Weak law enforcement and government framework | Before | 1 | 2 | 3 | 4 | 5 |
|  | During | 1 | 2 | 3 | 4 | 5 |
| 1. High demand of wild product and agricultural produce | Before | 1 | 2 | 3 | 4 | 5 |
|  | During | 1 | 2 | 3 | 4 | 5 |
| 1. Limited recognition of the value of biodiversity and environmental services | Before | 1 | 2 | 3 | 4 | 5 |
|  | During | 1 | 2 | 3 | 4 | 5 |
| 1. Others……………………. | Before | 1 | 2 | 3 | 4 | 5 |
|  | During | 1 | 2 | 3 | 4 | 5 |

1. Activities to address deforestation and forest degradation

To what extend do you agree that the following items can address drivers of deforestation and forest degradation in your region?

| **Activities** | **Strongly disagree (1)** | **Disagree (2)** | **Neutral**  **(3)** | **Agree**  **(4)** | **Strongly agree**  **(5)** |
| --- | --- | --- | --- | --- | --- |
| 1. Reinforcement of land-tenure | 1 | 2 | 3 | 4 | 5 |
| 1. Formulation of land-use plans (participatory land use planning) | 1 | 2 | 3 | 4 | 5 |
| 1. Community-based forest protection | 1 | 2 | 3 | 4 | 5 |
| 1. Implementation of Assisted Natural Regeneration (ANR) activities | 1 | 2 | 3 | 4 | 5 |
| 1. Fire prevention and control | 1 | 2 | 3 | 4 | 5 |
| 1. Introduction of fuel-efficient stoves | 1 | 2 | 3 | 4 | 5 |
| 1. Protection of livestock | 1 | 2 | 3 | 4 | 5 |
| 1. Agricultural intensification | 1 | 2 | 3 | 4 | 5 |
| 1. Water resource development projects | 1 | 2 | 3 | 4 | 5 |
| 1. NTFPs development activities | 1 | 2 | 3 | 4 | 5 |
| 1. Restoration | 1 | 2 | 3 | 4 | 5 |
| 1. Reduce forest crime through direct law enforcement (patrolling, monitoring system etc.) | 1 | 2 | 3 | 4 | 5 |
| 1. Establish community-based ecotourism | 1 | 2 | 3 | 4 | 5 |
| 1. Provide infrastructure support linked to conservation activities | 1 | 2 | 3 | 4 | 5 |
| 1. Develop and manage a system to share carbon benefits | 1 | 2 | 3 | 4 | 5 |
| 1. Improve literacy and numeracy | 1 | 2 | 3 | 4 | 5 |
| 1. Others……………………….. | 1 | 2 | 3 | 4 | 5 |

**Part 3. Local livelihood assessment ( before and during REDD+ implementation )**

**Natural capital:**

1. Situation of biodiversity (birds, animals, glass, water, fish, forest etc.)

**Before REDD+** : ☐ 1. Significantly decrease ☐2. Slightly decrease

☐ 3. Remain the same ☐ 4. Slightly increase ☐5. Significantly Increase

**During REDD+:** ☐1. Significantly decrease ☐ 2. Slightly decrease

☐ 3. Remain the same ☐4. Slightly increase ☐5. Significantly Increase

1. The improvement of forest coverage: Has the forestland coverage increase?

**Before REDD+** : ☐1. Significantly decrease ☐2. Slightly decrease

☐ 3. Remain the same ☐4. Slightly increase ☐5. Significantly Increase

**During REDD+:** ☐1. Significantly decrease ☐2. Slightly decrease

☐3. Remain the same ☐4. Slightly increase ☐5. Significantly Increase

1. The situation of forest protection

**Before REDD+** : ☐1. Very bad ☐ 2. Bad ☐3. Average

☐4. Good ☐5. Very good

**During REDD+:** ☐1. Very bad ☐ 2. Bad ☐3. Average ☐4. Good ☐5. Very good

1. The frequency of illegal logging and encroachment occurrences in community forest per month (in case there is no occurrence per month, as further for per year, and notice )

**Before REDD+** : ☐1.Never ☐2. Rarely (or once to twice) ☐3. Sometimes (three to five times) ☐4. Often (more than five times) ☐5. Very often (more than 10 times)

**During REDD+:** ☐1.Never ☐2. Rarely (or once to twice) ☐3. Sometimes (three to five times) ☐4. Often (more than five times) ☐5. Very often (more than 10 times)

1. Has REDD+ changed the traditional consumption mode of extracting forest ( ex. The way of harvesting wood products) ?

☐1.Extremely no ☐2. No ☐3. Neutral ☐4. Yes ☐5. Definitely yes

Explain:………………………………………………………………………………………………………………………………………………………………………

**Physical capital:**

1. Is there the increasing of household fix assets such as land, furniture, radio, telephone motor, boat etc. in your house?

**Before REDD+** : ☐1. Significantly decrease ☐2. Slightly decrease ☐3.​ Remain the same ☐4. Slightly increase ☐5. Significantly increase

**During REDD+:** ☐1. Significantly decrease ☐2. Slightly decrease ☐3.​ Remain the same ☐4. Slightly increase ☐5. Significantly increase

1. Is there the improvement of local utilities (water, electricity) ?

**Before REDD+** : ☐1. Significantly decrease ☐2. Slightly decrease ☐3.​ Remain the same ☐4. Slightly increase ☐5. Significantly increase

**During REDD+:** ☐1. Significantly decrease ☐2. Slightly decrease ☐3.​ Remain the same ☐4. Slightly increase ☐5. Significantly increase

1. Is there the improvement of infrastructure (roads, schools, health centers, dams, transportation)

**Before REDD+** : ☐1. Significantly decrease ☐2. Slightly decrease ☐3.​ Remain the same ☐4. Slightly increase ☐5. Significantly increase

**During REDD+:** ☐1. Significantly decrease ☐2. Slightly decrease ☐3.​ Remain the same ☐4. Slightly increase ☐5. Significantly increase

**Human capital:**

1. Is there the support for local production systems, including technical assistance and agricultural inputs for soil mechanization and irrigation?

**Before REDD+:** ☐1. Absolutely no ☐2. No ☐3. Neutral ☐4. Yes ☐5. Definitely yes

**During REDD+** : ☐1. Absolutely no ☐2. No ☐3. Neutral ☐4. Yes ☐5. Definitely yes

1. Is there the technical assistance for new enterprises (fish farming, poultry and beekeeping)?

**Before REDD+:** ☐1. Absolutely no ☐2. No ☐3. Neutral ☐4. Yes ☐5. Definitely yes

**During REDD+** : ☐1. Absolutely no ☐2. No ☐3. Neutral ☐4. Yes ☐5. Definitely yes

1. Is there any environmental education training conducted for local community or any training related to forest management or REDD+ per year?

**Before REDD+** : ☐1.Never ☐2. Rarely (or once to twice) ☐3. Sometimes (three to five times) ☐4. Often (more than five times) ☐5. Very often (more than 10 times)

**During REDD+:** ☐1.Never ☐2. Rarely (or once to twice) ☐3. Sometimes (three to five times) ☐ 4. Often (more than five times) ☐ 5. Very often (more than 10 times)

**Financial capital:**

1. How much you earn from related forest income (NTFPs collecting, fee from protecting forest etc.) per month?

**Before REDD+**: ☐1. Less than 10 USD ☐2. 11 to 25 USD

☐3. 26 to 50 USD ☐ 4. 51 to 100 USD

☐ 5. 101 to 200 USD ☐6. 201 to 300 USD

☐7. More than 300 USD

**During REDD+:** ☐1. Less than 10 USD ☐2. 11 to 25 USD

☐3. 26 to 50 USD ☐ 4. 51 to 100 USD

☐ 5. 101 to 200 USD ☐6. 201 to 300 USD

☐7. More than 300 USD

1. How much you earn from other activities beside forest related income (fishing, remittance, livestock, plantation, labor work) per month?

**Before REDD+**: ☐1. Less than 50 USD ☐2. 51 to 100 USD

☐3. 101 to 200 USD ☐ 4. 201 to 300 USD

☐ 5. 301 to 400 USD ☐6. 401 to 500 USD

☐7. More than 500 USD

**During REDD+**: ☐1. Less than 50 USD ☐2. 51 to 100 USD

☐3. 101 to 200 USD ☐ 4. 201 to 300 USD

☐ 5. 301 to 400 USD ☐6. 401 to 500 USD

☐7. More than 500 USD

1. Any resources change before REDD+ and during REDD+ development?

If yes: Explain the change ……………………………………………………… …………………………………………………………… …………… ………… …………………………………………………………………………………………………………………. …………………………………………… …………

If No: Explain the similarity ……………………………………………………… …………………………………………………………… …………… ………… …………………………………………………………………………………………………………………. …………………………………………… …………

1. Is there any limitation or restriction in accessing to land use ?

**Before REDD+** : ☐ 1. Very restriction ☐2. Slightly restriction ☐3. Neutral ☐4. Less restriction ☐5. No restriction

**During REDD+:** ☐ 1. Very restriction ☐2. Slightly restriction ☐3. Neutral ☐4. Less restriction ☐5. No restriction

1. Is there the improvement of agricultural production?

**Before REDD+** : ☐1. Significantly decrease ☐ 2. Slightly decrease ☐ 3. Neutral ☐4. Slightly improve ☐5. Significantly improve

**Please describe**: the agricultural activities…………………………………………..

……………………………………………………………………………………

……………………………………………………………………………………

**During REDD+:** ☐1. Significantly decrease ☐2. Slightly decrease ☐ 3. Neutral ☐4. Slightly improve ☐5. Significantly improve

**Please describe**: the agricultural activities…………………………………………..

………………………………………………………………………………………

………………………………………………………………………………………

**Social capital:**

1. Can you control over the resources (forestry production, NTFPs, ….)?

**Before REDD+** : ☐1. Very restriction ☐2. Restriction ☐3. Neutral

☐4. Control ☐5. Fully control

**During REDD+:** ☐ 1. Very restriction ☐2. Restriction ☐ 3. Neutral

☐4. Control ☐5. Fully control

1. Can you access to information related to REDD+ management ?

**Before REDD+** : ☐1. Absolutely No ☐ 2. No ☐3. Neutral ☐ 4. Yes ☐5. Absolutely Yes

**Please describe**: what information they know……………………………………… …..… …… ……… …… … …………… …………………………………….…

……………………………………………………………………………………

**During REDD+:** ☐1. Absolutely No ☐ 2. No ☐3. Neutral ☐ 4. Yes ☐5. Absolutely Yes

**Please describe**: what information they know……………………………………… …..… …… ……… …… … …………… …………………………………….…

……………………………………………………………………………………

1. Can you access to information about budget of REDD+ implementation?

**Before REDD+** : ☐1. Absolutely No ☐ 2. No ☐3. Neutral ☐ 4. Yes ☐5. Absolutely Yes

**Please describe**: what information they know……………………………………… …..… …… ……… …… … …………… …………………………………….

……………………………………………………………………………………

**During REDD+:** ☐1. Absolutely No ☐ 2. No ☐3. Neutral ☐ 4. Yes ☐5. Absolutely Yes

**Please describe**: what information they know……………………………………… …..… …… ……… …… … …………… …………………………………….

……………………………………………………………………………………

1. Can you access to information about planning of REDD+ implementation?

**Before REDD+** : ☐1. Absolutely No ☐ 2. No ☐3. Neutral ☐ 4. Yes ☐5. Absolutely Yes

**Please describe**: what information they know……………………………………… …..… …… ……… …… … …………… …………………………………….…

……………………………………………………………………………………

**During REDD+:** ☐1. Absolutely No ☐ 2. No ☐3. Neutral ☐ 4. Yes ☐5. Absolutely Yes

**Please describe**: what information they know……………………………………… …..… …… ……… …… … …………… …………………………………….…

……………………………………………………………………………………

1. Do you involve or participate in decision making about natural resources development or management?

**Before REDD+** : ☐1.Never ☐2. Rarely (or once to twice) ☐3. Sometimes (three to five times) ☐4. Often (more than five times) ☐5. Very often (more than 10 times)

**During REDD+:**  ☐1.Never ☐2. Rarely (or once to twice) ☐3. Sometimes (three to five times) ☐4. Often (more than five times) ☐5. Very often (more than 10 times)

1. Do you participate in any meeting​ for community or natural resources development and management ?

**Before REDD+** : ☐1.Never ☐2. Rarely (or once to twice) ☐3. Sometimes (fourth to five times) ☐4. Often (more than five times) ☐5. Very often (more than 10 times)

**During REDD+:** ☐1.Never ☐2. Rarely (or once to twice) ☐3. Sometimes (fourth to five times) ☐4. Often (more than five times) ☐5. Very often (more than 10 times)

1. How do you think about the situation of land tenure and right over land use?

**Before REDD+:** ☐1. Absolutely bad ☐2. Bad ☐3. Average ☐4. Good ☐ 5. Absolutely good

**During REDD+** : ☐1. Absolutely bad ☐2. Bad ☐3. Average ☐4. Good ☐ 5. Absolutely good

**Part 4: Local perception on the effectiveness of REDD+ development**

1. Does forest improve local people’s livelihood?

**Before REDD+:** ☐1. Absolutely no ☐2. No ☐3. Neutral ☐4. Yes ☐5. Definitely yes

**During REDD+** : ☐1. Absolutely no ☐2. No ☐3. Neutral ☐4. Yes ☐5. Definitely yes

1. Are you satisfied with your income from forest or natural related source?

**Before REDD+:** ☐ 1. Extremely no ☐2.No ☐3. Neutral ☐4. Yes ☐5. Definitely yes

**During REDD+:** ☐ 1. Extremely no ☐2.No ☐3. Neutral ☐4. Yes ☐5. Definitely yes

1. Are you satisfied with your income from other alternative source beside forest?

**Before REDD+:** ☐ 1. Extremely no ☐2.No ☐3. Neutral ☐4. Yes ☐5. Definitely yes

**During REDD+:** ☐ 1. Extremely no ☐2.No ☐3. Neutral ☐4. Yes ☐5. Definitely yes

1. What is your opinion on the infrastructure (roads, schools, health centers, dams, transportation) ?

**Before REDD+:** ☐1. Extremely poor ☐ 2.Poor ☐3. Neutral ☐4.Good ☐5. Definitely good

**During REDD+:** ☐1. Extremely poor ☐2.Poor ☐3. Neutral ☐ 4.Good ☐5. Definitely good

1. What is your perception of the ecological quality (forest, wildlife , NTFPs , water etc)? **Before REDD+:** ☐1. Very poor ☐2. Poor ☐3. Neutral ☐4. Good ☐5.Very good

**During REDD+:** ☐1. Very poor ☐2. Poor ☐3. Neutral ☐4. Good ☐5.Very good

1. Has your community integrated their planning and budget efficiency?

**Before REDD+:** ☐1. Extremely no ☐ 2. No ☐3. Neutral ☐4. Yes ☐5. Definitely yes

**During REDD+:** ☐ 1. Extremely no ☐2. No ☐3. Neutral ☐4. Yes ☐5. Definitely yes

1. How are you feeling about the collaboration and partnership with your community?

**Before REDD+** : ☐1. Extremely poor ☐2. Poor ☐3. Neutral ☐4. Good ☐5. Very good

**During REDD+:** ☐1. Extremely poor ☐ 2. Poor ☐3. Neutral ☐4. Good ☐5. Very good

1. Does your community always inspect/monitor the daily/weekly/monthly forest protection?

**Before REDD+:** ☐1. Extremely no ☐2. No ☐ 3. Neutral ☐4. Yes ☐5. Definitely yes

**During REDD+:** ☐1. Extremely no ☐2. No ☐3. Neutral ☐ 4. Yes ☐5. Definitely yes

1. Does your community always settle the matter promptly?

**Before REDD+:** ☐1. Extremely no ☐ 2. No ☐3. Neutral ☐4. Yes ☐5. Definitely yes

**During REDD+:** ☐1. Extremely no ☐2. No ☐3. Neutral ☐4. Yes ☐5. Definitely yes

1. Is the forest management and conservation evaluated by outsiders (by government or private company or NGOs, just not by community themselves) monthly/yearly forest protection?

**Before REDD+:** ☐1. Extremely no ☐2. No ☐3. Neutral ☐4. Yes ☐5. Definitely yes

**During REDD+:** ☐1. Extremely no ☐2. No ☐3. Neutral ☐4. Yes ☐5. Definitely yes

1. Does local household bear the burden to take part in forest protection and conservation (such as contribute the membership fee, being forest rangers, take part in conservation by giving time, and labor etc)

**Before REDD+:** ☐1. Extremely no ☐2. No ☐ 3. Neutral ☐4. Yes ☐5. Definitely yes

**During REDD+:** ☐1. Extremely no ☐2. No ☐ 3. Neutral ☐4. Yes ☐5. Definitely yes

1. Are you satisfied with the benefit that you get from forest (including both monetary and non-monetary benefit)?

**Before REDD+:** ☐1. Extremely no ☐2. No ☐ 3. Neutral ☐4. Yes ☐5. Definitely yes

**During REDD+:** ☐1. Extremely no ☐2. No ☐ 3. Neutral ☐4. Yes ☐5. Definitely yes

1. Do you have opportunities to work in any task related to forest management and conservation (get paid) ?

**Before REDD+:** ☐1. Extremely no ☐2. No ☐ 3. Neutral ☐4. Yes ☐5. Definitely yes

**During REDD+:** ☐1. Extremely no ☐2. No ☐ 3. Neutral ☐4. Yes ☐5. Definitely yes

1. Does your community have proper utilization of the monthly allocation of funds?

**Before REDD+:** 1. Extremely no 2. No 3. Neutral 4. Yes 5. Definitely yes

**During REDD+:** 1. Extremely no 2. No 3. Neutral 4. Yes 5. Definitely yes

1. Does your community have published the account regularly?

**Before REDD+:** ☐1. Extremely no ☐2. No ☐ 3. Neutral ☐4. Yes ☐5. Definitely yes

**During REDD+:** ☐1. Extremely no ☐2. No ☐ 3. Neutral ☐4. Yes ☐5. Definitely yes

1. Do you know about the carbon finance (the carbon can be sold) ?

**Before REDD+:** ☐1. Extremely no ☐2. No ☐ 3. Neutral ☐4. Yes ☐5. Definitely yes

**During REDD+:** ☐1. Extremely no ☐2. No ☐ 3. Neutral ☐4. Yes ☐5. Definitely yes

1. Do you know about (how) the benefits sharing of REDD+ in your community?

**Before REDD+:** ☐1. Extremely no ☐2. No ☐ 3. Neutral ☐4. Yes ☐5. Definitely yes

**During REDD+:** ☐1. Extremely no ☐2. No ☐ 3. Neutral ☐4. Yes ☐5. Definitely yes

1. What is the frequency of committee and assembly meeting (per year)?

**Before REDD+:** ☐1.Never ☐2. Rarely (or once to twice) ☐3. Sometimes (three to five times) ☐4. Often (more than five times)

☐5. Very often (more than 10 times)

**During REDD+**: ☐1.Never ☐2. Rarely (or once to twice) ☐3. Sometimes (three to five times) ☐4. Often (more than five times)

☐5. Very often (more than 10 times

1. Are you satisfied with the opportunities for decision-making and capacity building?

**Before REDD+:** ☐ 1. Extremely no ☐2. No ☐3. Neutral

☐4. Yes ☐5. Definitely yes

**During REDD+:** ☐ 1. Extremely no ☐2. No ☐3. Neutral

☐4. Yes ☐5. Definitely yes

1. How is the relationship between your community and the local government, NGOs and others?

**Before REDD+:** ☐1. Extremely poor ☐2. Poor ☐3. Neutral

☐4. Good ☐5. Very good

**During REDD+:** ☐1. Extremely poor ☐2. Poor ☐3. Neutral ☐4. Good ☐5. Very good

1. How do you feel about overall forest management and protection ?

**Before REDD+:** ☐1. Extremely poor ☐2. Poor ☐3. Neutral

☐4. Good ☐5. Very good

**During REDD+:** ☐1. Extremely poor ☐2. Poor ☐3. Neutral ☐4. Good ☐5. Very good

1. Are you willing to support forest management and protection ?

**Before REDD+:** ☐1. Extremely no ☐2. No ☐3. Neutral

☐4. Yes ☐5. Absolutely yes

**During REDD+:** ☐1. Extremely no ☐2. No ☐3. Neutral

☐4. Yes ☐5. Absolutely yes

1. Do you think the REDD+ development in your area is in the effective way ( long term benefit)? Why? Why not? ……………………………………………………. ………………………………………………………………………………………………………………………………………………………………………… ………………………………………………………………………………………..
2. Do you think what should be the appropriate strategies for effective forest management and protection in the future for your community? ……………………………………………………………………………………………………………………………………………………………………………

Thanks for your time and collaboration!
